# A Comparison of Bayesian Spatial Models for HIV Mapping in South Africa

**DOI:** 10.3390/ijerph182111215

**Published:** 2021-10-26

**Authors:** Kassahun Abere Ayalew, Samuel Manda, Bo Cai

**Affiliations:** 1 School of Mathematics, Statistics and Computer Science, University of KwaZulu-Natal, Pietermaritzburg 3209, South Africa; Samuel.Manda@mrc.ac.za; 2 Biostatistics Unit, South African Medical Research Council, Pretoria 0001, South Africa; 3 Department of Statistics, University of Pretoria, Pretoria 0028, South Africa; 4 Department of Epidemiology and Biostatistics, Arnold School of Public Health, University of South Carolina, Columbia, SC 29208, USA; BOCAI@mailbox.sc.edu

**Keywords:** Bayesian, disease mapping, skew-t distribution, ICAR-normal, ICAR-Laplace, spatial random effects, spatial model

## Abstract

Despite making significant progress in tackling its HIV epidemic, South Africa, with 7.7 million people living with HIV, still has the biggest HIV epidemic in the world. The Government, in collaboration with developmental partners and agencies, has been strengthening its responses to the HIV epidemic to better target the delivery of HIV care, treatment strategies and prevention services. Population-based household HIV surveys have, over time, contributed to the country’s efforts in monitoring and understanding the magnitude and heterogeneity of the HIV epidemic. Local-level monitoring of progress made against HIV and AIDS is increasingly needed for decision making. Previous studies have provided evidence of substantial subnational variation in the HIV epidemic. Using HIV prevalence data from the 2016 South African Demographic and Health Survey, we compare three spatial smoothing models, namely, the intrinsically conditionally autoregressive normal, Laplace and skew-t (ICAR-normal, ICAR-Laplace and ICAR-skew-t) in the estimation of the HIV prevalence across 52 districts in South Africa. The parameters of the resulting models are estimated using Bayesian approaches. The skewness parameter for the ICAR-skew-t model was not statistically significant, suggesting the absence of skewness in the HIV prevalence data. Based on the deviance information criterion (DIC) model selection, the ICAR-normal and ICAR-Laplace had DIC values of 291.3 and 315, respectively, which were lower than that of the ICAR-skewed t (348.1). However, based on the model adequacy criterion using the conditional predictive ordinates (CPO), the ICAR-skew-t distribution had the lowest CPO value. Thus, the ICAR-skew-t was the best spatial smoothing model for the estimation of HIV prevalence in our study.

## 1. Introduction

Governments in sub-Saharan Africa (SSA), in collaboration with non-governmental organizations and private sectors, design national strategic plans and policies, allocate resources and implement programs in the fight against the HIV/AIDS epidemic [1,2]. Such efforts are designed to reduce HIV-related infection, morbidity and mortality. As well as understanding the level of the HIV epidemic at the national level, most governments in the region have implemented a decentralized approach to governance and service provision. Thus the need for reliable local (district)-level HIV statistics to support decision making regarding the delivery of HIV care, treatment and prevention services [3,4]. Most of the countries in SSA rely on data obtained from national HIV surveys for monitoring the level of the HIV epidemic and subsequence responses. However, the national HIV surveys are mostly empowered to produce reliable HIV estimates at national and provincial level. Crude HIV estimates at small area level could be exaggeratedly estimated due to small numbers, resulting in unstable variances [5,6,7]. Consequently, HIV prevention and treatment programs tailored to small areas could be based on unreliable evidence [8].

As a result, modelling approaches are used for generating local-level estimates from survey data that are originally meant to provide reliable estimates at national and provincial levels [9,10]. The most used approach has been using spatial smoothing models where spatial components are incorporated in the model as random effects. The spatial models produce reliable disease rates with improved accuracy for small areas with few sparse observations by incorporating information from local, spatially contiguous areas. The structured random effect in spatial models represents clustering of diseases over geographical areas, unobserved environmental or frailty factors which are spatially correlated but are not included as covariates in a model [11,12,13]. Structured spatial random effects (which consider the local effects) are mostly modelled using the intrinsic conditional autoregressive normal (ICAR-normal) model (Besag et al [13], Carlin and Banerjee [14]). The ICAR-normal model offers greater flexibility for modelling the spatial correlation than the linear mixed effects model, with only a global random effect. However, a normal spatial distribution on the structured spatial effect could be restrictive, as there could be a possibility that the normality assumption could be misspecified [15]. Misspecification of the distribution of the random effects may result in estimates of diseases rates that are biased [16,17]. The usual approach is to transform the data to normality, for example by performing a logarithm of the rates. However, if there was an appropriate theoretical model, transformation could be avoided, as it is difficult to interpret results from transformed data. In addition, the transformation could result in the loss of information [17].

A few approaches have been proposed to reduce the impact of a normal distribution assumption for spatial random components. For example, Lunn et al [18] and Manda [19] proposed a double exponential and a mixture of ICAR-normal and ICAR-double exponential, respectively, to better capture possible wider tails for the spatial random effects. Kim and Mallick [20] and Azzalini and Capitanio [21] considered a skew-normal spatial model for point referenced data. However, the structured spatial skewed random fields suffer identifiability problems (since the skewness parameter may be unknown) [22] and must be determined uniquely [23]. To solve this identifiability problems, Zhang and El-Shaarawi [24] defined a skewed stationary Gaussian process for spatial random effect based on the work by Azzalini and Capitanio [21]. In addition, Allard and Naveau [25] and Zareifard and Jafari Khaledi [26] introduced a skew-normal spatial random field based on Domınguez-Molina et al [27] and Palacios and Steel [28], respectively, for point referenced data. Other skewed spatial distributions are the skew-normal by Rantini et al [29] and Fernández and Steel [30].

Our aim, in this study, is to model the district-level HIV prevalence in South Africa using spatial smoothing methods. There is ample evidence of substantial small area variation in the distribution of HIV prevalence in Sub-Saharan Africa [31,32]. Similarly evidence has also been found in South Africa by Kim et al [33] and Gutreuter et al [34]. The distribution of the district HIV prevalence could be skewed and non-normal. Thus, we estimated the spatial distribution of the HIV prevalence among the districts in South Africa using the ICAR-normal [13], ICAR skew-t distribution (Nathoo and Ghosh [35]) and ICAR-Laplace [18] using the 2016 South African Demographic and Health Survey data. The next section presents the description of the spatial models used and the HIV data. Section 3 contains the results obtained from fitting the models to the data. We discuss the results in Section 4 and conclude in Section 5.

## 2. Methods and Data Source

### 2.1. Skew-t Spatial Random Effects Distribution

Let $Y_{i}$ be the number of HIV positive individuals out of a sample of size $n_{i}$ in district i (i=1,…,52). Both $Y_{i}$ and $n_{i}$ are adjusted to account for the survey design to become the effective number of HIV cases, $Y_{i}^{*}$, and the effective sample size, $n_{i}^{*}$ [35,36,37,38]. A three-stage Bayesian hierarchical spatial smoothing model for a binary HIV outcome uses a binomial distribution at stage one as

$$Y_{i}^{*}|p_{i}\sim Binomial\left(n_{i}^{*},p_{i}\right),\;i=1,\ldots ,52$$

where $p_{i}$ is the proportion (prevalence) of HIV in district i and is modelled at the second stage by a logit link function using a set of district-level predictor variables, $X_{i}$, and both unstructured and spatially structured random effects, as introduced by Besag et al. (1991).

$$\log\left(\frac{p_{i}}{1-p_{i}}\right)=\beta_{0}+X_{i}\beta +u_{i}+v_{i}$$

where $\beta_{0}$ is the intercept; β is a vector of regression coefficients for predictor variable in $X_{i}$; $u_{i}$ is the unstructured random component and it is assumed to follow a normal distribution, $u_{i}\sim N\left(0,\;\sigma_{u}^{2}\right)$; $v_{i}$ is the structured spatial random component for district i.

The structured spatial random effects could be modelled using an intrinsic conditional autoregressive normal (ICAR-normal) prior (Besag et al [13], Knorr-Held and Best [12] and Carlin and Banerjee [14]) as

$$\;v_{i}|v_{-i}\sim ICARN\left(\mu_{v},\;\sigma_{v}^{2}\right)=N\left(\frac{\Sigma_{j\sim i}v}{m_{i}},\;\frac{\sigma_{v}^{2}}{m_{i}}\right)$$

where $m_{i}$ is the number of neighbours of district i. Lunn et al [18] suggested an alternative model based on a Laplace/double exponential distribution (ICAR-Laplace), which is given as $u_{i}\sim ICARL\left(\mu_{u},\;\sigma_{u}^{2}\right)$.

However, in situations where the distribution of HIV prevalence data could be non-normal and asymmetric, alternative spatial smoothing models that are robust and flexible could fit the data better. As a result, Nathoo and Ghosh [35] suggested the skew-t (ICAR-skew-t) spatial smoothing model, defined as

$$v_{i}|v_{-i}\sim ST_{v}\left(\frac{\Sigma_{j\sim i}v_{j}}{m_{i}},\;\frac{\sigma_{v}^{2}}{m_{i}},\;\delta_{v}\right)$$

For easy implementation in most Bayesian statistical software, Sahu et al [39] presented a suitable representation of skew-t distribution with k degrees of freedom. Suppose y~skew−t (k), then it could be expressed as $y=\eta^{-\frac{1}{2}}\left(∆\left|X_{0}\right|+X\right)$, where $X_{0}\sim N\left(0,1\right)$, $X\sim N\left(\mu ,\sigma^{2}\right)$, ∆ is the skewness parameter and $\eta \sim gamma\left(\frac{k}{2},\frac{k}{2}\right)$. The hierarchical set-up of this stochastic representation can be given as $Y/w\sim \;N\left(\mu +∆w,\frac{\Sigma }{\eta }\right)$, where $\left|X_{0}\right|=w\sim N\left(0,I_{k}\right)I(w>0)$. Thus, the ICAR-skew-t for the structured spatial random effect can be expressed as

$$v_{i}\sim N\left(\frac{\Sigma_{j\sim i}s_{j}}{m_{i}}+\delta_{v}w_{i},\frac{\sigma_{s}^{2}}{\eta *m_{i}}\right)$$

where $w_{i}\sim N\left(0,I\right)I(w_{i}>0)$, $s_{i/S_{-i}}\sim N\left(\frac{\Sigma_{j\sim i}s_{j}}{m_{i}},\;\frac{\sigma_{s}^{2}}{m_{i}}\right)$ and $\sigma_{s}^{2}$ and $\delta_{v}$ are the variance of $s_{i}$ and the skewness parameter, respectively. The hierarchical representation of the ICAR-skew-t model is shown in the Appendix A.

### 2.2. Methods for Comparing Competing Models

In this study, we used the deviance information criterion (DIC) and conditional predictive ordinates (CPO) for comparing models. The deviance information criterion was developed by Spiegelhalter et al [40] as a method used for comparing models in a Bayesian framework. It is a measure of a model’s goodness of fit or adequacy adjusted for a measure of model complexity measured as effective number of parameters. Let θ and $y=y_{1},\ldots ,y_{1}$ be the model parameter and data respectively, then DIC is expressed as

$$DIC=\bar{D}+p_{D}=2\bar{D}-D\left(\bar{\theta }\right)$$

where $\bar{D}=E_{\theta /y}\left[D\left(\theta \right)\right]=E_{\theta /y}\left[-2\log\;p\left(y/\theta \right)\right]$ and is the posterior mean deviance that measures the goodness of fit or adequacy $p_{D}=\bar{D}-D\left(\bar{\theta }\right)=E_{\theta /y}\left[D\left(\theta \right)\right]-D\left(E_{\theta /y}\left[\theta \right]\right)=E_{\theta /y}[-2\log\;p\left(y/\theta \right)]-[-2\log\;p\left(y/\bar{\theta }\left(y\right)\right]$ is a measure of the effective number of parameters and measures model complexity; larger values of $p_{D}$ suggests higher complexity of the model. It is also defined as the difference between the posterior mean of the deviance and the deviance at the posterior means of the parameters of interest; in other words, it is considered as the expected excess of the true residuals over the estimated residuals in the data conditional on the parameter θ [16]. Let $\theta^{1},\ldots ,\theta^{k}$ be parameter estimates from a converged Markov chain, then $\bar{D}$ is estimated as $\frac{1}{k}\overset{k}{\underset{1}{\sum }}D(\theta^{k})$ and $D\left(\bar{\theta }\right)=D(\frac{1}{k}\;\overset{k}{\underset{1}{\sum }}\theta^{k}$). 

The CPO is a leave-one-out cross validation approach that measures the posterior probability of observing $y_{i}$ when the model is fitted to all data excluding $y_{i}$ and it measures the predictive ability of the fitted model. Let$\;Y=Y_{1},\;Y_{2},\;\ldots ,\;Y_{n}$ be the nX1 data vector and $Y_{-i}$ be the data vector without $y_{i}$. Then, the conditional predictive ordinate for observation $y_{i}$ is given as

$$CPO_{i}=f\left(y_{i}/y_{-i}\right)=\int f\left(y_{i}/\theta \right)P\left(\theta /y_{-i}\right)d\theta =E_{\theta /y}\left[\frac{1}{f\left(y_{i}/\theta \right)}\right]$$

where θ is the parameter vector, $y_{i}$ is the ith observation and $y_{-i}$ is the observed data set except $y_{i}$. Thus, one can estimate the value of the inverse of $CPO_{i}$ by averaging the inverse probability function evaluated at $y_{i}$ for each $\theta^{k}$ produced from the posterior density. The $CPO_{i}$ values could be easily determined from the standard MCMC output which is given as

$$CPO_{i}={\left[\frac{1}{k}\;\overset{K}{\underset{k=1}{\sum }}\frac{1}{f\left(y_{i}/\theta^{k}\right)}\right]}^{-1}$$

which is the harmonic mean of the probability density function evaluated at $y_{i}$ for each $\theta^{k}$, where ***K*** is the number of iterations. For discrete data, the comparison of $CPO_{i}$ with the relative frequency determined from data without $y_{i}$ ($y_{-i}$) enables the assessment of the predictive capacity of the fitted model to the data. In order to compare two or more competing models, the overall CPO values of each model are assessed, given as $CPO=\underset{i}{\prod }CPO_{i}$; A model with higher CPO value suggests better predictive performance than the other models; hence, this model is preferred over other models. Mostly, the CPO value is close to zero, thus the negative of the sum of the log of the $CPO_{i}$ is used as indicated by Cai et al [41] and is given by $LS_{cv}=-\overset{k}{\underset{i=1}{\sum }}logCPO_{i}$. Thus, a model with the lowest $LS_{cv}$ value is the best model in terms of its predictive capacity.

### 2.3. Implementation

The model parameters were determined using a Bayesian estimation approach via Markov Chain Monte Carlo (MCMC) as implemented in OpenBUGS [42]. The prior distributions for the regression coefficients and the unstructured random component were the same for all the three models. The prior distribution for the intercept was $\beta_{0}\sim \mathrm{uniform}\;\mathrm{on}\;\left(-\infty ,\infty \right)$ and the prior for the regression coefficients was $\beta_{q}\sim N\left(0,\;0.00001\right)$, where q=1, 2, 3, 4; the variance parameters $\sigma_{u}^{2}$ and $\sigma_{v}^{2}$ were given as inverse gamma prior distributions with shape and scale parameters set at 20 and 2000, respectively. The skewness parameters for ICAR-skew-t were assigned $\delta_{v}\sim N\left(0,\;0.01\right)$ prior. We conducted a sensitivity analysis to determine the impact of the hyper-parameters of the priors on the outcome variable; for this, we chose the most commonly used hyper-parameters, such as IG(1000,1000), IG(10,10), IG(1,10)  and IG(2, 2000). Since prior distributions with larger variances are considered in the model, the estimates from this analysis are expected to be relatively robust. Moran’s I test was conducted on the model residuals to determine the presence of spatial correlation [43]. We ran 100,000 iterations for each model to make inferences. We determined the number of initial iterations that needed to be discarded by assessing the history plots of each model and for each parameter. Similarly, we also investigated the autocorrelation plots of each model and each parameter to determine the selection intervals to avoid correlation problems in the generated chains.

### 2.4. Data

The data analyzed were obtained from the 2016 South African Demographic and Health Survey (SADHS 2016). The SADHS 2016 was conducted for evaluating the country’s health programs by monitoring key milestones such as mortality, fertility, maternal and child health, nutrition, HIV, gender-based violence, etc. The data for measuring these indicators were collected by asking respondents relevant sociodemographic and behavioral characteristic questions and by collecting biological specimens. The SADHS 2016 survey employed a multistage stratified cluster sampling design to select households and/or respondents for the sample. All women between the age of 15 and 49 and men between the ages of 15 and 59 were included in the survey. Interview data were collected from a total of 8514 women and 3618 men and 6912 individuals were tested for HIV seropositivity. More information about SADHS 2016 can be obtained from the full study report [44].

**Figure 1:**
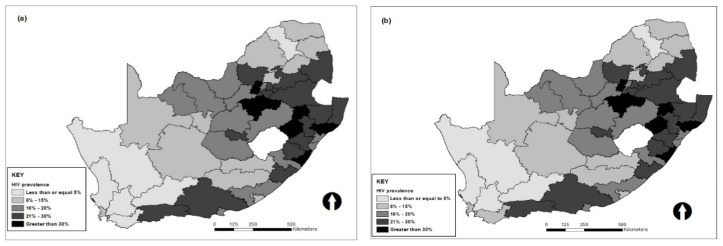
Map of HIV prevalence by district in South Africa before (**a**) and after (**b**) adjusting the data for zero positive tests in some districts.

The observed district-level HIV prevalence was computed by taking the survey design into account. The effective sample sizes in each district was determined by dividing the observed number of sample size at each district by the design effect [36]; the effective number of HIV cases is thus the product of effective sample size and the weighted prevalence. The number of HIV tests conducted in the survey by district varied substantially, with a sample size of between 8 tests and 455 tests, with a median sample size of 111 tests. There were some districts with zero count of HIV positive individuals in the sample. For this, we assigned them the average of the simulated data from a normal distribution with mean value equal to the average of the log of prevalence in the neighboring districts and variance as the variance of the log of the prevalence $p_{i}$ calculated from all the neighboring districts divided by the number of neighbors, shown in Figure 1b; the map in Figure 1a shows the raw data not adjusted for zero positive cases. A skewness test was conducted on the prevalence, with and without adjusting for zero HIV prevalence, but no significant skewness was found.

The covariates included in the models are the multidimensional poverty index constructed using the 2016 community survey data [45], HIV prevalence among pregnant women obtained from the 2017 National Antenatal Sentinel Survey report [46], population density and male condom distribution coverage obtained from the 2017 district health barometer report [47]. Previous studies indicate that these factors are associated with HIV prevalence ecologically as well as individually [3,48].

## 3. Results

The skewness parameters for ICAR-skew-t were not significant, perhaps suggesting that the spatial component is lighter tailed (see Table 1). The model with the lowest $LS_{cv}$ and DIC values was deemed to be the best model in its predictive performance and goodness of fit, respectively. Thus, as can be seen in Table 1, the model with the lowest $LS_{cv}$ (170.5) is the ICAR-skew-t model, followed by the ICAR-normal model ($LS_{cv}$= 172.4). The ICAR-normal model and the ICAR-Laplace model have the lowest (291.3) and second lowest (315) DIC values, respectively. The difference in the DIC values between these models is more than five, suggesting that there is substantial difference between the two models in terms of goodness of fit to the data, according Spiegelhalter et al [40]; however, a study by De la Cruz and Branco [49] indicated that DIC is not appropriate for such type of complex models. Thus, based on the $LS_{cv}$ values, the ICAR-skew-t model was the best in terms of its predicative capacity as compared to the other two models used in this study.

As a sensitivity analysis, we ran the analysis using different sets of hyper-parameters for priors of the precision parameters. Thus, the mean difference in the values of the outcome variables at different choices of hyper-parameter values was observed at the third digit after the decimal point, which suggests the absence of a significant impact on the outcome variable. The Moran’s I test statistic was significant (*p*-value = 0.000001), suggesting that residuals were spatially clustered. As shown in Table 1, district-level ANC prevalence is the strong predictor of district-level HIV prevalence determined from the 2016 SADHS data, whereas the other covariates were not statistically significant.

**Figure 2:**
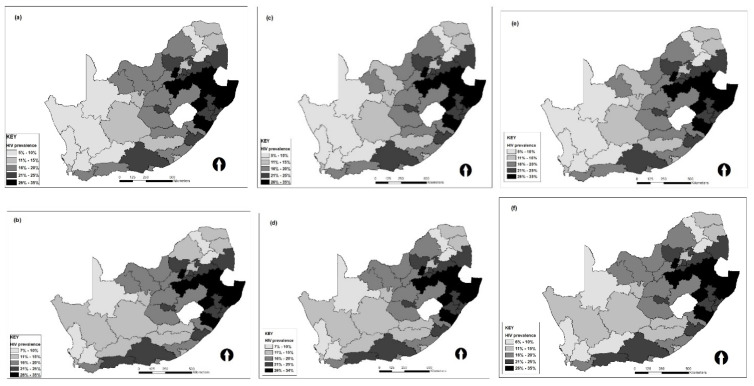
Estimated HIV prevalence by district in South Africa with covariates (first row **a**,**c**,**e**) and without covariates (second row **b**,**d**,**f**).

Figure 2e, shows the prevalence of HIV by district in South Africa estimated using the ICAR-skew-t spatial model (best model). According to the estimates from this model, most of the districts with high levels of HIV prevalence are located in southeastern parts of the country, while low levels of HIV prevalence are in the southwestern parts. This pattern is the same for all the maps produced using estimates from different models with or without covariates. Maps (a), (c) and (e) are estimates of the ICAR-normal, ICAR-Laplace and skew-t models with covariates, respectively; the spatial pattern of HIV prevalence is the same for these models, except the estimate from the ICAR-normal model for one district in the northwestern part. Maps (b), (d) and (f) are estimates of the ICAR-normal, ICAR-Laplace and skew-t models without covariates and the pattern of HIV prevalence by district is the same for the estimates determined using these models. One notable difference for the pattern of estimates with and without covariates for the models is that the level of HIV prevalence is lower for estimates with covariates than those without covariates in two districts in the western part.

## 4. Discussion

HIV is a leading cause of disease burden in sub-Saharan Africa. In the era of decentralized approach to governance and service provision, designing effective HIV intervention programs and monitoring strategies at local administrative levels requires reliable estimates of local variation in HIV burden. Our study compared three spatial smoothing models, namely, the intrinsically conditionally autoregressive normal, Laplace and skew-t (ICAR-normal, ICAR-Laplace and ICAR-skew-t) in the estimation of the HIV prevalence across 52 districts in South Africa. It analyzed HIV prevalence data from the 2016 South African Demographic and Health Survey. The models were fitted using the Markov Chain Monte Carlo method in OpenBUGS, a freely available Bayesian statistical package. We found that the ICAR-skew-t distribution was the best spatial smoothing model for the estimation of HIV prevalence in our study.

We found that the districts with high levels of HIV prevalence were in the southeastern parts of the country, while low levels of HIV prevalence corresponded to the southwestern parts. Our findings are similar to those by Gutreuter et al [34] and Woldesenbet et al [46]. The estimates of HIV prevalence by district in South Africa could help governmental and non-governmental originations, as well as the private sector, to know the level of the epidemics at lower administrative level, thus prioritizing and plan appropriate public health programs tailored to each community and evaluating the combined impact of national and local public health programs.

A major weakness of our study could be that there were no HIV data in some of the sparsely populated districts; hence, we simulated data from neighboring districts to estimate prevalence of HIV in such districts; thus, the estimates for these districts may not be reliable and should be interpreted with caution. In addition, a limited number of predictors was included in the model; hence, some important predictors of district-level HIV prevalence might be missing.

## 5. Conclusions

In conclusion, alternative spatial distributions to ICAR-normal should be considered for modeling spatial disease outcomes. The spatial random effects could be skewed or non-normal and misspecification of the distribution of random effects could lead to estimates that are biased. This could lead to implications in the estimation of disease burden, adversely impacting policy derivations. In our study, we found that the intrinsic conditional autoregressive skew-t (ICAR-skew-t) model was the best in predicting district-level HIV prevalence compared to the ICAR-normal and ICAR-Laplace spatial models based on an analysis of the 2016 South African Demographic and Health Survey (2016 SADHS) data. District antennal clinic HIV prevalence was the most influential predictor of the district-level 2016 SADH HIV prevalence.

## Figures and Tables

**Table 1:** Comparison of the fitted models using DIC and CPO.

Covariates	ICAR-Normal	ICAR-Laplace	ICAR-Skew-t
Intercept	2.473 (−3.288, −1.65)	−2.542 (−3.321, −1.743)	−2.538 (−3.625, −1.469)
Population density	−0.0001 (−0.0003, 0.0002)	−0.0001 (−0.0003, 0.0002)	0.0001 (−0.0003, 0.0002)
Male condom distribution	−0.0070 (−0.0183, 0.0069)	−0.0064 (−0.0178, 0.0039)	−0.0069 (−0.0177, 0.0032)
Multidimensional poverty index	0.81056 (−2.826, 4.7939)	0.593 (−3.139, 4.357)	0.8934 (−2.915, 4.71)
ANC HIV prevalence	3.778 (1.673, 5.7058)	3.974 (2.074, 5.897)	3.831 (1.7, 5.931)
$\sigma_{v}^{2}$	0.0061 (0.0006, 0.6596)	0.0059 (0.0006, 0.9225)	0.0088 (0.0009, 0.4719)
$\sigma_{u}^{2}$	0.0066 (0.0007, 0.2281)	0.0106 (0.0011, 0.2434)	0.0031 (0.0004, 0.1688)
$\delta_{u}$			0.05 (−0.6, 0.62)
DIC	291.3	315	348.1
$LS_{cv}$	172.4	174	170.5

## Data Availability

The dataset used in this study are available from the Demographic and Health Survey (DHS) website https://dhsprogram.com/Data/ (accessed on 1 August 2021) upon request from the MEASURE DHS program team.

## Appendix A

Hierarchical representation of the disease mapping model presented in Section 2.1, assuming the spatial random components follows skew-t distribution is given as follows

$$Y=Y_{1},\;Y_{2},\;\ldots ,\;Y_{n}\;$$

be a one-dimensional random variable with binomial distribution

$$logit\;(p_{i})=\beta_{0}+X_{i}\beta +u_{i}+v_{i}\;u_{i}\sim N\left(0,\;\sigma_{u}^{2}\right)\;v_{i/}S_{i},\sigma_{v}^{2},\delta_{v},w_{i},\sim N\left(\frac{\Sigma_{j\sim i}u_{j}}{m_{i}}+\delta_{u}w_{i},\;\frac{\sigma_{s}^{2}}{\eta *m_{i}}\right)\;s_{i/S_{-i}}\sim N\left(\frac{\Sigma_{j\sim i}s_{j}}{m_{i}},\;\frac{\sigma_{s}^{2}}{m_{i}}\right)\;w_{i}\sim N\left(0,I\right)I(w_{i}>0)\;\eta \sim gamma\left(\frac{k}{2},\frac{k}{2}\right)\;\beta_{i}\sim N\left(\beta_{0,}\Lambda \right),\;i=0,1,2,\;\ldots ,\;k$$

where *k* is the number of covariates

$$\sigma_{v}^{2}\sim IG\left(\Omega ,v\right)\;\delta_{u}\sim N\left(0,\;\Gamma \right)\;\sigma_{s}^{2}\sim IG\left(\Omega ,u\right)\;k\sim Exp\left(k_{0}\right)I(k>2)$$

where $p_{i}$ is the weighted prevalence corresponding to $Y_{i}$ i=1, 2, …, 52, $\sigma_{u}^{2}$ and $\sigma_{v}^{2}$ are variance of the spatial and the heterogeneous random component, $I(w_{i}>0)$ is an indicator function, IG is inverse gamma and Exp is exponential.

Based on the likelihood distribution and the above prior specifications the posterior distribution of all the parameters assuming conditional independence between the response variable and the hyper parameters is given as

$$p\left(\mu ,\beta ,\;u,v,\sigma_{u}^{2},\sigma_{v}^{2},\delta_{u},w,k,\eta ,s/y\right)\propto L\left(y/\beta ,\;u,v,\sigma_{s}^{2},\sigma_{v}^{2},\delta_{u},w,s\right)P\left(\beta ,\;u,v,\sigma_{s}^{2},\sigma_{v}^{2},\delta_{u},w,k,\eta \right)\;=\prod_{i}p(y_{i}/\mu_{i})\prod_{j}(p(\beta_{j}/\Lambda )p\left(\Lambda \right))p\left(u/\sigma_{s}^{2}\right)p\left(\sigma_{s}^{2}\right)p\left(v/\sigma_{v}^{2}\right)p\left(\sigma_{v}^{2}\right)p\left(s/\sigma_{s}^{2}\right)p\left(w\right)p\left(\delta_{u}\right)p\left(k\right)p\left(\eta \right)$$
